# The CX3CL1-CX3CR1 axis in mesenchymal stem cell regulation and bone diseases

**DOI:** 10.3389/fendo.2026.1992376

**Published:** 2026-10-30

**Authors:** Ziyun Kuang, Yixin Zhang, Jiao Li

**Affiliations:** School of Basic Medical Sciences, Zunyi Medical University, Zunyi, China

**Keywords:** BMSCs, bone remodeling, CX3CL1, CX3CR1, osteoporosis

## Abstract

The chemokine axis CX3CL1-CX3CR1 is involved in the whole process of bone remodeling, bone injury repair, and inflammation-mediated bone destruction, making it both a diagnostic marker and a potential target for therapy. This article focuses on bone marrow mesenchymal stem cells (BMSCs) to explain the bidirectional mechanism of CX3CL1-CX3CR1 in regulating bone homeostasis, compares three types of targeting strategies, and proposes a local delivery approach. The regulatory effect of CX3CL1 is concentration-dependent: transient or moderate sCX3CL1 signaling is associated with a pro-repair macrophage phenotype, while persistent pathological elevation promotes M1 polarization and bone resorption; however, direct evidence for the low intramedullary concentration of the M2 axis is limited. This article focuses on analyzing the hormone regulatory pathways of three endocrine-related bone diseases (postmenopausal osteoporosis, diabetic bone disease, and hyperthyroidism-induced bone injury), and compares the advantages and disadvantages of monoclonal antibodies, small molecule drugs, and bone nanocarriers. The CX3CL1-CX3CR1 axis represents a candidate research target for pre-clinical investigations into endocrine-related bone diseases, yet substantial unresolved barriers limit its translational progression. Local targeted delivery strategies may offer conceptual directions for future pre-clinical investigation for solving the treatment challenges brought about by the bidirectional effects of this pathway, but their clinical feasibility still needs to be verified. Notably, critical in-vivo concentration thresholds distinguishing protective versus pathological CX3CL1 effects have not been established, and evidence supporting concentration-dependent M1/M2 macrophage polarization is largely derived from non-bone systems.

## 1 Introduction

In recent years, with the acceleration of population aging and changes in lifestyle, the incidence of bone diseases such as osteoporosis, osteoarthritis, and impaired fracture healing has been increasing year by year, and the age of onset is becoming younger. This has brought a serious burden to people’s lives and the healthcare industry. The pathogenesis of bone diseases is highly complex, involving multiple biological processes such as innate immune inflammation, stem cell differentiation imbalance, and disruption of bone resorption and formation homeostasis. BMSCs are the core matrix cells that maintain bone metabolism balance and mediate bone injury repair. As precursor cells of osteoblasts, they have a certain impact on bone formation efficiency. Paracrine chemokines and cytokines regulate the polarization and activation of bone marrow macrophages and osteoclast precursors, serving as a critical link between bone matrix cells and immune cells (1–5). The chemokine family mediates intercellular communication in the bone marrow microenvironment and plays an irreplaceable role in regulating BMSC migration, proliferation, and differentiation. Among them, the CX3CL1-CX3CR1 signaling axis has become a research hotspot in bone immunology and stem cell regeneration in recent years. CX3CL1, a unique transmembrane molecule in the CX3C chemokine family, pairs with its specific receptor CX3CR1 to form a complete signaling axis, with dual functions: membrane-bound (mCX3CL1) mediates short-range cell adhesion communication, and soluble cleaved product (sCX3CL1) mediates long-distance chemotactic cell migration (6). The chemokine signaling network has been proven to be a core regulatory hub of skeletal homeostasis. CX3CL1-CX3CR1, together with other chemokine axes (such as CCL2/CCR2, CCL5/CCR5), participates in the balanced regulation of bone formation and resorption (7). The main types of chemokines in bone tissue include CCL2, CCL4, and CCL8, which are secreted by osteoblasts and play key roles in bone development and structural maintenance. Previous reviews mostly focused on a single disease or single intervention, such as Wojdasiewicz on osteoporosis, Tanaka on rheumatoid arthritis, and Chen on MSC-derived extracellular vesicle strategies for treating osteoporosis (8–10). They generally lack an integrated perspective that links the full spectrum of bone diseases with BMSCs as the core under endocrine metabolic bone diseases, and seldom provide quantitative thresholds for the bidirectional regulation of CX3CL1 concentration or analyze the safety issues caused by the bidirectional effects of targeting pathways. This article reviews from three levels: molecular interactions, pathogenesis of endocrine metabolic bone diseases, and targeted translational strategies, with a focus on postmenopausal osteoporosis, diabetic bone disease, and hyperthyroidism-related bone damage. It clarifies the concentration-dependent regulation patterns of CX3CL1, compares the three intervention strategies, and provides theoretical support for basic and translational research on endocrine-related bone diseases.

## 2 The interaction mechanism between the CX3CL1-CX3CR1 axis and BMSCs

### 2.1 Chemokines are key mediators of signal communication between cells in the bone marrow microenvironment

CX3CL1, different from the CC and CXC chemokine subfamilies, is the only transmembrane protein in the CX3C branch, and it has dual biological activities as both a cell adhesion molecule and a soluble chemokine. Two key metalloproteinases, ADAM10 and ADAM17, are responsible for its cleavage, and they differ markedly in subcellular localization, substrate affinity, and activation triggers (11–14). ADAM10 mediates constitutive cleavage, predominantly located within membrane lipid-raft domains. Under physiological conditions, ADAM10 executes low-level, continuous shedding, maintaining basal concentrations of soluble CX3CL1 (sCX3CL1) without strong inflammatory stimulation (15–18). In contrast, ADAM17 (also known as TACE) localizes to non-lipid-raft membrane compartments and mediates inducible cleavage. Its activity remains quiescent at resting state but is robustly activated upon inflammatory or stress stimuli, generating large quantities of sCX3CL1 with much higher catalytic efficiency than ADAM10 (19–21). This “constitutive-inducible” division of labour between the two proteases constitutes a fine-tuning switch for CX3CL1 bioavailability. mCX3CL1 retained on cell surfaces mediates short-range cell-to-cell adhesion. Once cleaved, diffusible sCX3CL1 is released into the extracellular space to exert long-range chemotactic activity (20). At the transcriptional level, the osteogenic master transcription factor RUNX2 directly represses *ADAM17* transcription by binding to its gene promoter region, forming a molecular link between bone-formation signalling and CX3CL1 shedding machinery (22, 23). Jones and colleagues revealed that in rheumatoid synovium, ADAM17, p38 MAPK, and cathepsins collaboratively regulate CX3CL1 synthesis and release (24). Beyond CX3CL1 processing, ADAM10 and ADAM17 also participate in negative-feedback regulation of osteoclast differentiation independent of CX3CL1 cleavage (25, 26). Metalloproteinase inhibitors can selectively block either constitutive ADAM10-dependent or inducible ADAM17-dependent shedding, providing experimental tools to dissect mCX3CL1- versus sCX3CL1-driven signalling (20).

### 2.2 The bidirectional regulatory mechanism of CX3CL1-CX3CR1 on BMSCs

Under resting conditions, BMSCs mainly express membrane-bound mCX3CL1, which maintains the basal proliferation activity of the cells and the homeostasis of the bone marrow microenvironment. When inflammatory stimuli such as TNF-α, IL-1β, and LPS are present, the intracellular NF-κB signaling pathway is activated, significantly upregulating the transcription of the *CX3CL1* gene and increasing the total expression level of CX3CL1 in the cells (27, 28). Estrogen and androgen can regulate the expression levels and activation states of the ADAM17 protease in bone marrow mesenchymal stem cells and bone marrow stromal cells, thereby altering the ratio of mCX3CL1 to sCX3CL1, and indirectly regulating the intensity of the downstream CX3CR1 signaling pathway. The dysregulation of this regulatory pathway is considered to be an upstream potential trigger for postmenopausal osteoporosis and osteoporosis associated with thyroid dysfunction (10). After the inflammatory injury occurs, ADAM17 is highly activated, prompting BMSCs to release high levels of soluble sCX3CL1 into the bone marrow microenvironment; sCX3CL1 can attract CX3CR1^+^ macrophages and osteoclast precursor cells to migrate towards the injury site, thereby forming a local chemotactic microenvironment centered on BMSCs (15). The CX3CL1-CX3CR1 axis achieves its bidirectional functions in the bone microenvironment through a spatiotemporal dynamic regulatory network. The differential cleavage of CX3CL1 by ADAM10/17 is the core regulatory node of this network (23).

#### 2.2.1 CX3CL1-CX3CR1 regulates the proliferation and growth cycle of BMSCs

Hao L et al. constructed a *CX3CL1* silencing lentiviral vector and transfected it into rat BMSCs. The CCK-8 experiment results showed that cell proliferation was significantly inhibited 48 hours after *CX3CL1* silencing; flow cytometry for cell cycle detection confirmed that knockdown of *CX3CL1* could cause BMSCs to be arrested in the S phase, and the expression of proliferation-related protein PCNA was synchronously downregulated (29). At the mechanism level, endogenous *CX3CL1* in BMSCs can bind to its own low-expression CX3CR1 through autocrine manner, activating the ERK1/2 pathway to promote the expression of cyclins. Exogenous sCX3CL1 can dose-dependently restore the proliferation ability of BMSCs, and this rescue effect completely disappears after blocking CX3CR1, confirming that CX3CR1 is the essential receptor for CX3CL1 to regulate the proliferation of BMSCs (30). The gene expression profiling analysis revealed that the expression of *CX3CL1* in BMSCs showed dynamic changes during osteogenic induction, suggesting that CX3CL1 might increase *Runx2* transcription by activating the p38 MAPK pathway and thereby participate in the regulation of osteogenic differentiation of BMSCs (31).

#### 2.2.2 CX3CL1 concentration-dependent regulation of osteogenic and adipogenic differentiation of BMSCs

The core feature of bone marrow metabolic imbalance is the weakened osteogenic differentiation of BMSCs and the enhanced adipogenic differentiation. The CX3CL1-CX3CR1 axis can bidirectionally regulate this differentiation balance, and its biological effects are significantly dependent on concentration and microenvironment. Based on available *in vitro* cell experiments and *in vivo* bone injury models, accumulating evidence suggests that low concentrations of sCX3CL1 may promote BMSC osteogenic differentiation via the CX3CR1-dependent MAPK/ERK pathway, accompanied by increased expression of Runx2, ALP, and osteocalcin. This effect potentially facilitates tissue mineralization and callus formation, which may contribute to bone injury repair (32, 33). On the contrary, under pathological conditions, continuous high concentrations of sCX3CL1 will excessively activate the NF-κB signaling pathway, directly inhibiting the transcriptional activity of Runx2, while upregulating the key transcription factor *PPARγ* for adipogenic differentiation, promoting BMSCs to differentiate preferentially into adipocytes; long-term imbalance will lead to a significant accumulation of fat in the bone marrow cavity, accompanied by a decrease in the number of bone trabeculae and bone density, participating in the pathological process of osteoporosis (34, 35). *In vitro* experiments showed that recombinant sCX3CL1 could exert osteogenic effects within the concentration range of 10 to 50 ng/mL. The baseline concentration of CX3CL1 in healthy human peripheral serum was only 20 to 60 pg/mL, a difference of thousands of times. It is speculated that in the damaged bone marrow area, sCX3CL1 may be locally enriched, reaching nanogram levels locally. However, the local concentration of this bone marrow cannot be directly detected in the human body. Currently, there is no established critical concentration threshold in the body that can distinguish between protective effects and pathological effects. The level of peripheral serum CX3CL1 cannot be directly equated with the actual ligand concentration in the bone marrow microenvironment (33, 36). Different studies have reported that there are certain differences in the baseline levels of serum CX3CL1 in healthy individuals. In this cohort, the serum CX3CL1 level of postmenopausal osteoporosis patients was 139.8 ± 44.3 pg/mL, significantly higher than the level of the healthy control group in the same study. The serum CX3CL1 concentration of postmenopausal osteoporosis patients was positively correlated with the severity of the disease, and could be used as a potential biomarker for assessing the risk of bone loss (36). Studies have confirmed that in pathological microenvironments such as estrogen deficiency, high sugar levels, and radiation, BMSCs can undergo excessive adipogenic differentiation, which is one of the core pathogenic mechanisms of osteoporosis and diabetic bone diseases (37–39). Based on current research, it is speculated that the signaling characteristics of mCX3CL1 and the cleavage product of sCX3CL1 are significantly different: mCX3CL1 only initiates mild and short-range signals at the cell contact interface; sCX3CL1 can diffuse to form a concentration gradient and remotely regulate CX3CR1-positive cells, with the signal dynamics depending on the local ligand concentration. This “short-range adhesion long-range chemotaxis” signal diversion model constitutes an important molecular basis for the concentration-dependent bidirectional regulation of the CX3CL1-CX3CR1 axis (15, 40).

#### 2.2.3 CX3CL1 mediates the interaction between BMSCs and macrophages, as well as osteoclasts

It should be emphasized at the outset that existing evidence linking sCX3CL1 concentration gradients to macrophage M1/M2 polarization is predominantly derived from non-bone tissues; direct in-situ experimental evidence within the bone marrow-BMSC microenvironment remains sparse. Macrophage polarization status is also shaped jointly by multiple local cytokines and disease stages, rather than by CX3CL1 concentration alone. With these caveats in mind, a hypothetical working model has been proposed as follows:

The sCX3CL1 secreted by BMSCs can specifically attract CX3CR1-positive mononuclear macrophages and osteoclast precursor cells. It regulates the polarization balance of macrophages in both directions, thereby influencing the process of bone remodeling (10, 25). During different stages of bone injury repair, the polarization of macrophages and the concentration of CX3CL1 exhibit a dynamic change pattern: In the early stage of injury (1-3 days after injury), a small amount of sCX3CL1 attracts M1-type macrophages to infiltrate the site, mediating the clearance of necrotic tissue and initiating the inflammatory response (41). During the proliferation phase (3-7 days after injury), the concentration of sCX3CL1 gradually increases, and M1 type macrophages transform into M2 type, secreting anti-inflammatory factors such as TGF-β and IL-10, promoting the proliferation and osteogenic differentiation of BMSCs. In the bone remodeling phase (7-14 days after injury), sCX3CL1 reaches its peak and then decreases, with M2 type macrophages dominating, mediating bone callus mineralization and bone tissue remodeling (42, 43). The CX3CL1-CX3CR1 signaling axis can directly regulate osteoclast differentiation. The chemokine receptor CX3CR1 plays a dual role in maintaining the bone homeostasis of mice, participating in the maintenance of bone homeostasis by regulating the balance of osteoblast and osteoclast differentiation. Most supporting observations come from extra-skeletal systems, and analogous studies performed directly in bone tissue are scarce (**Figure 1**).

**Figure 1:**
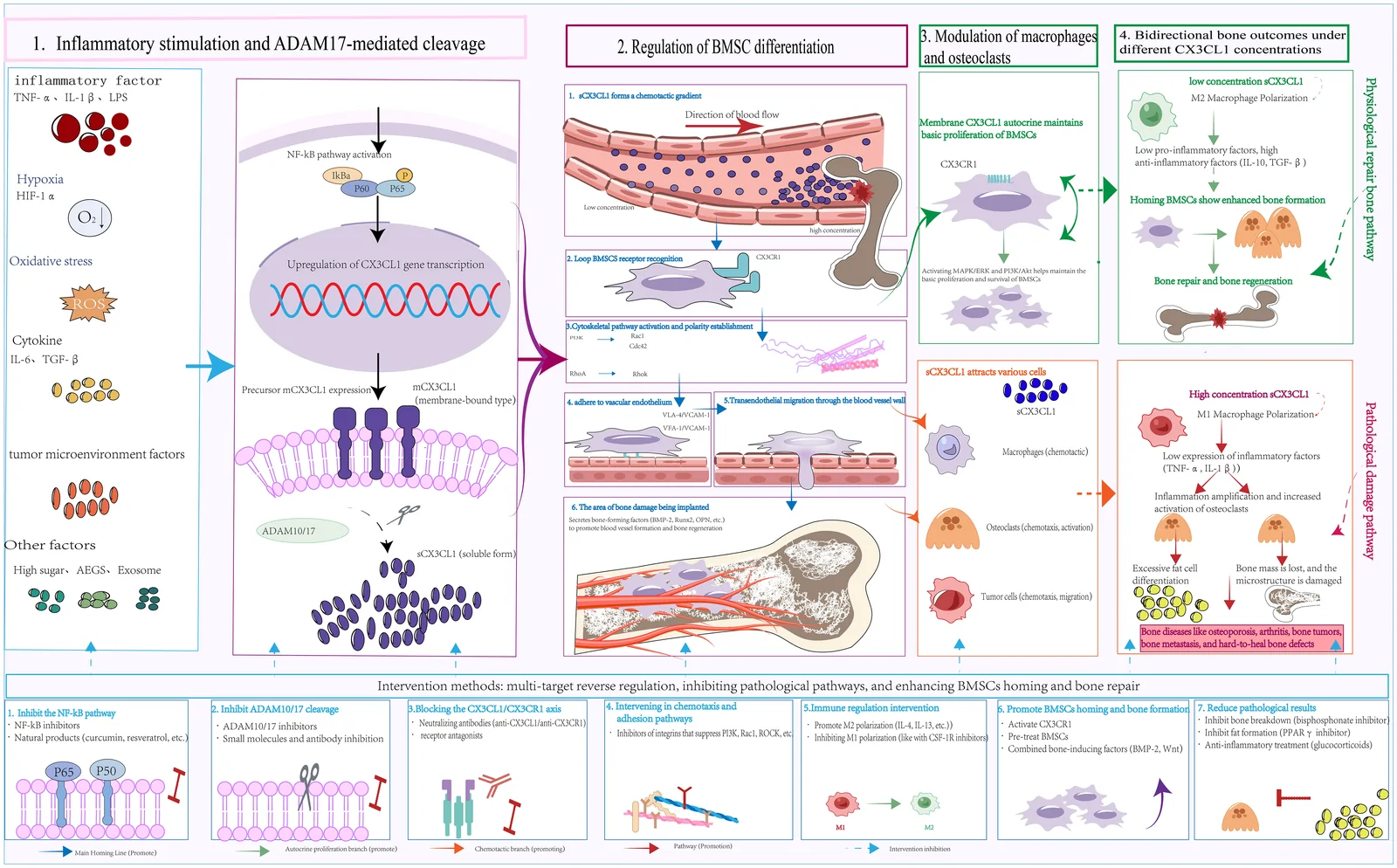
Schematic diagram illustrating the mechanism by which the CX3CL1-CX3CR1 axis regulates the interaction between BMSCs and cells in the bone marrow immune microenvironment. This figure illustrates the molecular mechanism by which the CX3CL1-CX3CR1 signaling axis mediates the interactions between BMSCs and various cells in the bone marrow microenvironment. Module 1 (Inflammatory stimulation and ADAM17 cleavage): TNF-α, IL-1β, etc. stimulate the activation of the NF-κB and ERK pathways within BMSCs, synergistically upregulating the transcription of *ADAM17*, and the activated ADAM17 cleaves mCX3CL1 to release sCX3CL1 (15). ADAM17 was first identified as a major protease mediating PMA-induced cleavage of CX3CL1 (14). ADAM10 appears to mediate constitutive low-level cleavage to maintain basal sCX3CL1 levels under physiological conditions (16, 20). ADAM10/17 may negatively regulate osteoclast differentiation via cleavage of RANKL and M-CSF receptors (26). Module 2 (Regulation of BMSC Differentiation): *In vitro* experiments demonstrated that low concentrations of sCX3CL1 (10-50 ng/mL) activated ERK/RUNX2, promoting osteogenic differentiation of BMSCs; whereas sustained high sCX3CL1 levels activated NF-κB, upregulated PPARγ, and promoted adipogenic differentiation (34, 35). These concentrations are supraphysiological and may not reflect local levels in the bone marrow niche. Module 3 (Macrophage/Osteoclast Regulation): In the early stage of injury, low levels of sCX3CL1 may induce local infiltration of M1-type macrophages, which could mediate necrotic tissue clearance and initiate inflammatory responses. M1 macrophages can secrete RANKL to promote osteoclast differentiation (42). CX3CL1 may also drive M1 polarization and osteoclast differentiation via NSUN5-mediated m5C RNA methylation modification (41).I It may also directly promote the differentiation of CD16^-^ monocytes into osteoclasts. Module 4 (Bidirectional Concentration Outcomes): It is hypothesized that low local sCX3CL1 levels help preserve bone homeostasis, while excessive sCX3CL1 may contribute to bone destruction. The red arrow indicates the pro-inflammatory/pro-resorptive pathway, and the green arrow indicates the osteogenic/anti-inflammatory pathway. This figure represents a purely hypothetical working model. To reiterate, direct in-bone evidence for concentration-dependent M1/M2 switching downstream of sCX3CL1 is currently lacking, and macrophage phenotypes are modulated by the broader local cytokine milieu beyond CX3CL1-CX3CR1 signaling alone.

## 3 Roles of the CX3CL1-CX3CR1 axis in BMSC-mediated bone pathologies

### 3.1 Postmenopausal osteoporosis

Postmenopausal osteoporosis is a typical endocrine-metabolic bone disease caused by estrogen deficiency, which leads to imbalance in bone metabolism. Estrogen exerts its bone-protective effect mainly through estrogen receptor α (ERα): 17β estradiol regulates the biological activities of osteoblasts and osteoclasts via ERα; in the absence of estrogen (such as during menopause), the balance between bone resorption and bone formation shifts towards increased bone resorption (44–47). Estrogen deficiency upregulates the activity of the CX3CL1-CX3CR1 axis through the following pathways: (1) ERα, as a nuclear transcription factor, can directly regulate the transcription of the *ADAM17* gene. During estrogen deficiency, the inhibitory effect of ERα on the transcription of ADAM17 is weakened, leading to an increase in ADAM17 expression; (2) Estrogen deficiency enhances the activity of the NF-κB signaling pathway. The NF-κB p65 subunit binds to the NF-κB binding site in the ADAM17 promoter region, synergistically upregulating the transcription of *ADAM17* (15, 48). (3) After the activity of ADAM17 is enhanced, it extensively cleaves mCX3CL1 to release sCX3CL1, thereby creating a local high concentration chemotactic gradient in the bone marrow (14). The serum CX3CL1 level in postmenopausal osteoporosis patients is significantly higher than that in healthy individuals. Clinical tests revealed that the peripheral serum CX3CL1 level in patients with PMOP can reach 139.8 ± 44.3 pg/mL, while the baseline concentration in healthy premenopausal women is approximately 109.7 ± 19.4 pg/mL, and that in postmenopausal non-osteoporosis women is approximately 116.5 ± 23.1 pg/mL (36). Studies have shown that the level of serum CX3CL1 is negatively correlated with the bone mineral density (BMD) of the lumbar spine and femoral neck, and positively correlated with the bone resorption marker TRACP5b; serum with high concentration of CX3CL1 is positively correlated with the bone turnover markers TRACP5b, NTx, and the inflammatory factors IL-1β and IL-6 (10, 36, 49). Ovarian oestrogen deprivation-induced oestrogen deficiency can upregulate the expression of CX3CL1 in the bone marrow of mice. The MicroCT results confirmed that the complete knockout of *CX3CR1* could significantly improve the number of bone trabeculae and bone volume fraction in OVX mice, and alleviate the bone loss induced by oestrogen deficiency (50). The ERα-specific knockout mouse model further indicates that the absence of ERα in BMSCs can upregulate the expression of *ADAM17*; based on the evidence of the molecular mechanism, it is speculated that the increase in ADAM17 can enhance the cleavage and release of membrane-bound CX3CL1, thereby inhibiting the osteogenic differentiation ability of BMSCs (44, 46).

### 3.2 Diabetic osteopathy

Diabetic osteopathy is a serious skeletal complication of type 2 diabetes (T2DM), characterized by impaired bone quality and increased risk of fractures. It is notable that the bone density of T2DM patients is often normal or even slightly elevated, indicating that the decline in bone quality is the core factor driving the increase in bone fragility (51–54). Potential upstream mechanisms include: chronic high glucose environment activates the NF-κB signaling pathway within BMSCs. Existing studies have confirmed that NF-κB can directly bind to the *CX3CL1* promoter and promote its transcription. Under high glucose conditions, NF-κB activation may mediate the increase in CX3CL1 expression in BMSCs. Further verification in the BMSCs system is needed (55–57). A high-sugar environment promotes the massive accumulation of advanced glycation end products (AGEs); when AGEs combine with their receptor RAGE, it induces an increase in cellular oxidative stress levels. Oxidative stress can act as an upstream signal to further enhance the transcriptional activity of NF-κB. Based on existing molecular evidence, it is speculated that this pathway ultimately promotes the upregulation of CX3CL1 expression; the AGERAGE signaling pathway can initiate a positive feedback loop where ROS and NF-κB mutually activate; this vicious cycle can continuously amplify inflammatory signals, and thus it is hypothesized that it can maintain the persistent high expression state of CX3CL1 (58). The high-sugar environment upregulates the expression and activity of ADAM17, promoting the extensive cleavage and release of mCX3CL1 into sCX3CL1 (59). The research has confirmed that the serum CX3CL1 level of patients with T2DM combined with osteoporosis is significantly higher than that of patients with T2DM alone and healthy controls. The increase in serum CX3CL1 level is associated with glycolipid metabolism disorders, abnormal bone metabolism, and decreased bone density, and it is a risk factor for osteoporosis in patients with T2DM (60, 61). The results of both population observation and *in vitro* experiments indicate that a high sugar environment can promote the expression of CX3CL1. Clinical studies have found that individuals with elevated glycated hemoglobin levels have higher concentrations of CX3CL1 in their circulation, suggesting that the state of blood sugar control may be involved in regulating the expression level of CX3CL1. However, further validation is still needed through large-sample correlation analysis on T2DM (62).

### 3.3 Hyperthyroidism-induced bone injury

In the state of hyperthyroidism, excessive thyroid hormones (T3 and T4) can directly act on bone tissue, accelerating the rate of bone turnover. The degree of bone resorption exceeds the rate of bone formation, ultimately leading to bone loss (63, 64). The potential molecular mechanism underlying the upregulation of ADAM17 expression by thyroid hormones: T3 can enhance the activity of the NF-κB signaling pathway; previous studies have confirmed that activating NF-κB can promote *ADAM17* transcription. After ADAM17 expression and activity increase, it can cleave mCX3CL1, promoting the release of sCX3CL1into the circulation. Clinical studies have found that the level of CX3CL1 in the blood of hyperthyroid patients is elevated and is accompanied by impaired bone quality, suggesting that the chemokine CX3CL1 may be an important factor connecting thyroid function status and the balance of bone remodeling; *in vitro* evidence shows that thyroid hormone membrane receptor signaling regulates CX3CL1 expression, and this effect can be antagonized by the thyroid hormone derivative tetrac, but its regulatory role in bone tissue still needs further verification (65). Studies have shown that the serum CX3CL1 level in patients with Graves’ disease (toxic diffuse thyroiditis) is significantly elevated, and it is negatively correlated with the trabecular bone score (TBS), suggesting that high CX3CL1 levels are closely related to the destruction of bone microstructure in patients with hyperthyroidism (66, 67).

### 3.4 Traumatic fractures and postoperative delayed bone healing

During the process of fracture repair, the CX3CL1-CX3CR1 pathway exhibits temporal dynamic regulatory characteristics: In the early stage of injury, a small amount of sCX3CL1 is released within the fracture hematoma, and chemotactic circulating and bone marrow-derived CX3CR1^^+^^ BMSCs migrate to the injury site and recruit M1-type macrophages to initiate tissue clearance. During the proliferative phase, the secretion of sCX3CL1 by BMSCs and local stromal cells gradually increases, inducing macrophages to polarize towards the M2 type, which then secretes factors such as TGF-β and IL10 to promote the osteogenic process. In the bone remodeling phase, the level of sCX3CL1 peaks and then decreases, and M2-type macrophages become dominant, promoting the mineralization of the bone callus (68). However, under the continuous stimulation of persistent chronic inflammatory conditions such as infection, diabetes, and severe soft tissue injuries, BMSCs continuously synthesize large amounts of CX3CL1, resulting in long-term high expression of sCX3CL1 in the local area. Excessive recruitment of M1-type macrophages to fracture ends amplifies the local inflammatory cascade, inhibits the osteogenic activity of BMSCs, hinders bone callus mineralization, and ultimately induces delayed fracture healing or even non-union of the bone. In osteoporotic fractures, *CX3CR1^+^* inflammatory osteoclasts (iOCs) abnormally increase in the bone callus. By secreting Sema3A, they inhibit the axonal regeneration of CGRP^^+^^ TrkA^^+^^ sensory neurons and the neural-bone signal exchange, thereby hindering the bone remodeling process. Targeting *CX3CR1^^+^^* iOCs can restore sensory nerve signals and accelerate bone callus remodeling (69). Clinical evidence indicates that patients with poor fracture healing after trauma have significantly elevated serum CX3CL1 levels, and CX3CL1 is negatively correlated with bone metabolism protective indicators such as calcium and 25(OH)D__3__ (70). The combined detection strategy of markers is helpful in improving the predictive efficacy of fracture healing disorders; the predictive value of the combined assessment of CX3CL1 and Sema3A is superior to that of a single indicator, and the elevated CX3CL1 is an independent risk factor for delayed fracture healing and nonunion of bones (71, 72). Treadmill training can inhibit the secretion of CX3CL1, reduce the differentiation of osteoclasts, and promote tendon-bone healing, providing a new theoretical basis for fracture rehabilitation (73).

### 3.5 Knee osteoarthritis

Chronic low-grade inflammation of the synovium in knee osteoarthritis (KOA) is the core upstream trigger that distinguishes it from simple mechanical degenerative diseases. The affected synovium continuously releases pro-inflammatory factors such as IL-1β and TNF-α; the inflammatory signals can spread to the subchondral bone marrow microenvironment, continuously stimulating bone marrow and subsynovial bone BMSCs to upregulate CX3CL1 expression; at the same time, the inflammatory microenvironment enhances the activity of ADAM17 protease, promoting the extensive cleavage and release of membrane-bound CX3CL1 into the local microenvironment as soluble sCX3CL1 (14, 74, 75). Through bioinformatics analysis and experimental verification, it was confirmed that *CX3CR1* is a core gene related to OA, and it is closely associated with lipid metabolism, extracellular matrix dynamics, and immune cell infiltration (76). A two-way two-sample Mendelian randomization study confirmed a causal association between the elevated genetic prediction of circulating CX3CL1 levels and an increased risk of osteoarthritis (*OR* = 1.070, 95% CI: 1.040–1.110; *P* < 0.010), providing genetic evidence for the involvement of CX3CL1 in the pathogenesis of OA (77). Furthermore, *circPVT1* can promote the progression of OA through the *miR-550a-3p*/CX3CR1 axis. CX3CR1, as a key node in the competitive endogenous RNA network, participates in the regulation of chondrocyte growth, apoptosis, release of inflammatory mediators, and extracellular matrix metabolism (78). The researchers further confirmed that CX3CR1 is an important chemotactic factor-related biomarker for OA through Mendelian randomization and transcriptome analysis (79).

### 3.6 Ankylosing spondylitis bone erosion

Based on the validation using the SKG mouse AS model and clinical peripheral blood and intestinal tissue samples, it was found that the expression of CX3CL1 in AS patients was significantly upregulated throughout the body. This pathway mediates the systemic bone metabolism disorder of the “intestinal-bone inflammatory axis” (38, 80). The inflammatory mediators produced by chronic intestinal inflammation can enter the circulatory system and act on the bone marrow microenvironment. *In vitro* experiments have confirmed that pro-inflammatory signals can stimulate bone marrow BMSCs to synthesize and release sCX3CL1; excessive sCX3CL1 can attract monocytes to differentiate into M1 macrophages. Activated M1 macrophages secrete a large amount of RANKL, which further induces excessive differentiation of osteoclasts, ultimately causing local bone erosion in the sacroiliac joint and vertebral bodies of the spine, and triggering systemic osteoporosis (81–83). The above-mentioned reaction is considered to be an important potential mechanism for the bone metabolic disorder mediated by the “intestinal-bone inflammation axis” in ankylosing spondylitis.

### 3.7 Other bone diseases

#### 3.7.1 Radiation-induced bone damage

The radiation mouse model confirmed that ionizing radiation stimulates the endothelial cells of bone blood vessels and the bone marrow BMSCs to highly express CX3CL1 (38). The molecular mechanism is as follows: Ionizing radiation activates the HIF-1α/VEGF-A pathway; HIF-1α binds to the promoter of ADAM17 and activates the HRE element to upregulate the transcription of ADAM17; ADAM17 extensively cleaves mCX3CL1 to release sCX3CL1, while simultaneously upregulating CXCL2/CXCL12 to form a positive feedback amplification loop, attracting CD11b^+^ osteoclast precursor cells to aggregate, and causing a sharp increase in bone resorption (38, 84, 85). This mechanism can serve as a universal molecular model for the induction of CX3CL1 upregulation by stress.

#### 3.7.2 Tumor bone metastasis

The occurrence of bone metastasis in solid tumors such as prostate cancer and lung cancer is highly dependent on the CX3CL1-CX3CR1 signaling axis: The bone marrow microenvironment continuously secretes soluble CX3CL1 to form a chemotactic gradient, and cells expressing CX3CR1 in the circulating tumor cells migrate along the concentration gradient, achieving homing to the bone marrow and thereby completing the colonization of bone metastasis. The sCX3CL1 secreted by BMSCs in the bone marrow microenvironment forms a chemotactic gradient, attracting CX3CR1-positive tumor cells to colonize the bone marrow, thereby inducing bone metastasis foci. The specific regulatory signals (androgen-dependent, MAPK/ERK-dependent, etc.) of this pathway in different tumor types vary, but all have the CX3CL1-CX3CR1 chemotactic axis as the core link (28, 86–88, **Table 1**).

**Table 1:** Summary of serum CX3CL1 concentration ranges in healthy individuals and patients with various bone diseases.

Type of disease	Serum CX3CL1 concentration	Research population	References
Healthy pre-menopausal women	109.7 ± 19.4 pg/mL	Healthy control group	(10, 36)
Postmenopausal non-osteoporosis women	116.5 ± 23.1 pg/mL	Healthy post-menopausal women	(10, 36)
Postmenopausal osteoporosis (PMOP)	139.8 ± 44.3 pg/mL	PMOP patients	(10, 36, 38)
Type 2 diabetes mellitus combined with osteoporosis	Significantly higher than simple T2DM	Patients with T2DM and OP	(59)
Hyperthyroidism (Graves’ disease)	Significantly higher than the healthy control group	Patients with Graves’ disease	(67)
Delayed healing of traumatic fractures	Significantly higher than the normal healing group	Patients with delayed fracture healing	(71)
Poor prognosis of KOA	Significantly higher than the group with good prognosis	Patients with KOA	(102)

The concentration of sCX3CL1 in the local microenvironment of the bone marrow is not equivalent to that in the peripheral serum. The dosage of cell administration *in vitro* (10-50 ng/mL) cannot be directly equivalent to the detection values in human serum. There may be numerical differences among different ELISA kits and detection platforms. In clinical applications, a standardized detection system needs to be established.

## 4 Preclinical and emerging intervention strategies targeting the CX3CL1-CX3CR1 signaling axis

Currently, the targeted interventions targeting the CX3CL1-CX3CR1 pathway can be mainly classified into three categories: monoclonal antibodies, small molecule antagonists, and bone-targeted nano-delivery systems. Each strategy has different targets, administration methods, advantages, limitations, clinical stages, and applicable diseases (**Table 2**).

**Table 2:** Comparison of three intervention strategies targeting the CX3CL1-CX3CR1 signaling axis.

Intervention strategy	Target	Administration method	Main advantage	Main limitations	Clinical stage	Matching diseases
Monoclonal antibody (E6011/ 5H8-4)	sCX3CL1 (ligand)	Intravenous injection	High specificity, neutralizing cycle and local sCX3CL1	Systemic administration may interfere with the physiological repair pathways; the production cost is high.	Phase II Clinicaltrial of RA (E6011) (9); AS animal Experiments (41)	RA, AS, PMOP, KOA
Small molecule antagonist (AZD8797/ JMS-17-2)	CX3CR1 (receptor)	Oral administration/ Intracapsular injection	Can be taken orally, with convenient Administration; reversible binding	The Ki value of AZD8797 for CX3CR1 is 3.9 nM, and for CXCR2 it is 2800 nM. There is a CXCR2 off-target effect, which may interfere with the physiological inflammatory regulation and neutrophil recruitment mediated by CXCR2; hepatic and renal accumulation toxicity	KOA animal experiment (78), Animal experiments on osteoporosis; Clinical manifestations before tumor bone metastasis (103)	KOA, Osteoporosis, tumor bone metastasis
Bone-targeting nano-delivery system	Both ligands and receptors can	Intravenous injection/ Intra-articular injection	Targeted accumulation of the lesion, long-acting sustained- release, reducing systemic side effects	The *in vivo* biocompatibility needs to be verified; the problem of large-scale production arises	Animal Experiments (101)	Osteoporosis, KOA, Metabolic Bone Disease

### 4.1 Monoclonal antibody

Quetmolimab (KANAb001, E6011) is the world’s first humanized monoclonal antibody targeting CX3CL1. Its mechanism of action is to specifically neutralize sCX3CL1 in the circulation and local tissues, thereby inhibiting the infiltration of M1 macrophages into inflammatory tissues and blocking the abnormal activation of osteoclasts. It can simultaneously alleviate joint inflammatory responses and bone erosion damage (89–91). The mouse-specific CX3CL1 neutralizing antibody (clone 5H84) can be used for the intervention of rheumatoid arthritis. Intravenous injection of this antibody can effectively neutralize CX3CL1 in the bone marrow and local tissues, significantly reducing the recruitment of CD11b^+^ osteoclast precursor cells to the inflammatory bone tissue, and inhibiting abnormal bone remodeling. MicroCT bone microstructure detection confirmed that CX3CL1 blockade can effectively increase the number of bone trabeculae and bone volume fraction, while down-regulating bone resorption markers such as TRACP and βCTX, and inhibiting excessive bone resorption. In addition, as a key chemokine mediating bone injury in inflammation, the abnormal activation of CX3CL1 can participate in the disorder of the systemic inflammatory circuit. Targeted inhibition of CX3CL1 can effectively block the continuous bone destruction process driven by inflammation (25, 42, 92). Systemic administration of monoclonal antibodies can simultaneously neutralize the physiological low concentration of sCX3CL1, which may interfere with the early repair of bone damage. Bone-targeted carriers (such as HA nanoparticles) delivering monoclonal antibodies locally are expected to solve this contradiction. In addition, proteinase inhibitors targeting ADAM10/17 can differentially block the constitutive and inducible cleavage of CX3CL1, providing a new intervention strategy for regulating the CX3CL1 signal (19, 20, 93, 94).

### 4.2 Small molecule antagonists

AZD8797 (also known as KAND567) is a highly selective allosteric antagonist of CX3CR1 and belongs to a non-competitive, orally administrable small molecule compound (3, 95–97). *In vitro* experiments on OA chondrocytes demonstrated that after AZD8797 blocked CX3CR1, the activity of the NF-κB pathway decreased by more than 60% (76). The mechanism study indicates that the continuously activated CX3CR1 signaling pathway in the pathological microenvironment of osteoporosis can promote the chemotaxis and differentiation of osteoclast precursors, and drive excessive bone resorption. *In vitro* experiments have confirmed that the CX3CR1 selective antagonist AZD8797 can effectively block the formation of osteoclasts induced by CX3CL1 (10, 25, 73). The preclinical study of the novel selective CX3CR1 antagonist JMS-17-2 focused on the direction of tumor bone metastasis. It can inhibit the bone marrow homing of tumor cells by blocking the CX3CR1 pathway. Since the CX3CL1-CX3CR1 signal can promote the recruitment and differentiation of osteoclast precursor cells and exacerbate bone resorption, JMS-17-2 theoretically can simultaneously intervene in bone metastasis and bone loss (25, 28, 88, 98). However, the experimental results of AZD8797 and JMS-17-2 are all derived from preclinical studies. Whether these small molecules can simultaneously intervene in bone metastasis and bone loss in patients has not been reported yet and requires further research.

### 4.3 Nanotechnology and targeted drug delivery systems

The traditional systemic drug delivery strategies include neutralizing antibodies, small molecule CX3CR1 antagonists, and siRNA interference systems. However, they all have significant shortcomings: after intravenous administration, the drugs are difficult to effectively accumulate in the bone marrow cavity and the joint injury lesion sites, and it is difficult to achieve an effective intervention concentration locally (10, 99, 100). Bone-targeted nanocarriers can achieve the targeted enrichment of drugs in the active areas of bone remodeling and exert a long-term sustained-release effect. Currently, the mainstream carriers are mainly divided into three categories. (1) Exosome mimics modified with phosphatidylserine (PS-EMS) have been used to deliver CXCR3 antagonists in osteoporosis models, but there is still a lack of evidence for direct delivery targeting CX3CL1-CX3CR1. HA nanoparticles and gene nanocarriers are still in the early preclinical or theoretical stage. They may achieve local sustained release, but their dosing intervals, efficacy, and human safety still need to be verified. The pharmacological effect of AZD8797, a highly selective CX3CR1 antagonist, has been reported, and the combination strategy needs further verification (95, 101). (2) Hydroxyapatite (HA) nanoparticle bone-targeted delivery system. The HA crystals have the same structural pattern as the inorganic mineral lattice of human bone trabeculae and subchondral bone, and naturally possess the ability to specifically adsorb onto bone tissues. By loading anti-CX3CL1 neutralizing antibodies or CX3CR1 small molecule antagonists onto HA nanoparticles, this system can be used for local intervention in osteoporosis and knee osteoarthritis (KOA). After injection into the joint cavity, the carrier can prolong the retention time of the drug in the synovium and bone matrix, achieving long-term sustained release. Preclinical data indicate that this system can prolong the retention time of the drug in the local bone tissue, and it may enable a reduction in the frequency of administration. However, this conjecture still requires further verification through *in vivo* experiments (95, 101). At present, the nano HA-loaded chemoattractant-targeted preparations for joint cavity injection in KOA are still in the theoretical exploration stage. (3) Gene nano-delivery vector, which uses non-viral nano-systems to encapsulate *CX3CL1* siRNA, targets and silences the abnormally elevated *CX3CL1* in the bone marrow microenvironment of BMSCs. This approach avoids the inherent immunogenicity and genomic integration risks of viral vectors such as lentiviruses. It provides a theoretical basis for exploring long-term intervention strategies for metabolic osteoporosis related to type 2 diabetes and hyperthyroidism. However, its practical application still faces many technical bottlenecks (101). The strategy of encapsulating chemokine receptor antagonists using phosphatidylserine-modified exosome mimics has been reported in the literature. Compared to ordinary non-targeted liposomes, the active targeting effect mediated by PS can significantly enhance the enrichment efficiency of the nanocarriers in the bone remodeling area, and better inhibit the excessive activation of osteoclast lineage cells (101). However, there are no direct reports yet on the intervention of CX3CR1 antagonists targeting the CX3CL1 pathway by PS-EMs. The relevant protocols still need to be verified through experiments.

## 5 Summary and outlook

The CX3CL1-CX3CR1 chemokine axis serves as a critical osteo-immune hub governing BMSC proliferation, osteogenic-adipogenic differentiation, and cellular crosstalk with macrophages and osteoclast progenitors. In-vitro and pre-clinical animal data demonstrate distinct functions for membrane-bound mCX3CL1 and ADAM-cleaved soluble sCX3CL1. Aberrant activation of this pathway contributes to the pathogenesis of multiple metabolic and inflammatory bone diseases. Clinical observations show elevated circulating sCX3CL1 in affected patients, suggesting its potential biomarker value. Nevertheless, several major bottlenecks impede mechanistic understanding and translational progress. First, no validated in-vivo concentration threshold distinguishes protective versus pathological sCX3CL1 effects. In-vitro functional concentrations differ by orders of magnitude from human serum values, and peripheral sCX3CL1 cannot faithfully reflect actual ligand levels within the bone-marrow niche, which cannot be directly quantified in humans. Second, the concentration-dependent M1/M2 macrophage-polarization model is largely derived from non-bone systems; direct in-situ evidence within the BMSC-bone-marrow microenvironment remains scarce. Macrophage phenotypes are shaped by the full local cytokine milieu and disease status rather than CX3CL1 concentration alone. Third, existing therapeutic modalities including neutralizing antibodies, small-molecule antagonists and bone-targeted nanocarriers are confined to in-vitro and pre-animal studies. Human data regarding efficacy, safety and off-target risk are absent. Systemic intervention may disrupt physiological CX3CL1 signalling required for endogenous bone repair. Fourth, standardised biomarker detection protocols for CX3CL1 are lacking. Variability across ELISA platforms and sample-handling protocols limits cross-cohort comparability of clinical measurements. Future research should prioritize mechanistic experimental validation ahead of clinical translational attempts. New tools for spatially resolved quantification of intra-medullary sCX3CL1 are required to establish bone-relevant physiological concentration ranges. Bone-oriented co-culture and animal models are needed to resolve whether sCX3CL1 gradients independently drive macrophage phenotype switching in skeletal niches. Pre-clinical head-to-head comparisons between systemic and local bone-targeted delivery will help mitigate risks arising from the dual-edged nature of CX3CL1-CX3CR1 signalling. Multi-centre standardized cohorts are needed to define the clinical utility of circulating CX3CL1 as a prognostic or diagnostic biomarker. Targeting upstream ADAM10/ADAM17 proteases and MSC-derived extracellular vesicles also warrant further pre-clinical investigation. Only after these fundamental questions are addressed can the true translational potential of this axis for refractory bone disorders be properly evaluated.
